# Using 2% PVPI topical solution for serial intravitreous injections and ocular surface findings: a case control study

**DOI:** 10.1186/s40942-024-00557-1

**Published:** 2024-05-29

**Authors:** José Henrique Casemiro, Ana Paula Miyagusko Taba Oguido, Antonio Marcelo Barbante Casella

**Affiliations:** 1 Postgraduate Program in Health Sciences, State University of Londrina, UEL, Londrina, Brazil; 2 Department of Health Sciences, Surgical Clinic, State University of Londrina, UEL, Londrina, Brazil; 3 Londrina State University, Avenida Robert Koch, 60, Londrina, Paraná 86038-440 Brazil

**Keywords:** Dry eye disease, Intravitreal injections, Povidone iodine, Ocular surface disease

## Abstract

**Background:**

The use of povidone-iodine for ocular surface asepsis is widespread for intravitreal injections. They became frequent procedures, leading to serial exposure of patients’ eyes to iodinated solutions. In this study, we investigate the changes in the ocular surface in patients submitted to repeated use of povidine for intravitreal injection of anti-VEGF asepsis, analyzing Ocular Surface Disease Index, non-invasive break up time, blinking quality, lipid layer, meniscus height and osmolarity.

**Methods:**

This case-control study included 34 individuals (68 eyes), 14 males, 20 females aged 48 to 94. Inclusion criteria were individuals who received application of 2% povidone-iodine eyedrops for intravitreal injections treatment with the non-treated contralateral eye used as control. Ocular surface examinations were performed at a single occasion. A pre-intravitreal injection asepsis protocol with povidone-iodine was applied. All statistical analysis was performed using the STATA® 18.0 Software and a p-value = 0.05 was considered as the statistical significance value in all tests.

**Results:**

The median number of IVIs in treated eyes was 12 (range 6–20). The results in treated eyes compared with untreated eyes were respectively : median OSDI 16 (IQR 6–39) and 12.5 (IQR 8–39) (*p* = 0.380); mean NIBUT 10.30 (SD ± 2.62) and 10.78 (SD ± 2.92) ( s, *p* = 0.476); median blinking quality 100 (IQR 100) and 100 (IQR 100 ) (%, *p* = 0.188); median lipid layer 87 (IQR 77–90) and 86 (IQR 74–100) (nm, *p* = 0.451); median meniscus height 0.22 (IQR 0.19-0,31) and 0.24 (IQR 0.20–0.27) (mm, *p* = 0.862), median Meibomian gland atrophy 33 (IQR 24–45) and 31.5 (IQR 25–39) (%, *p* = 0.524); and mean osmolarity 306.6 (SD ± 21.13) and 313.8 (SD ± 29) (mOsm, *p* = 0.297). There was no statistically significant relationship between the repetitive use of 2% iodinated solution and signs or symptoms compatible with dry eye syndrome in this group of patients.

**Conclusions:**

The findings suggest that 2% povidone iodine is a safe and efficacious agent for ocular surface antisepsis during intravitreal injections, not leading to substantial ocular surface modifications. This conclusion supports the continued use of povidone iodine in routine ophthalmic procedures without increased risk of inducing dry eye syndrome.

## Background

The use of povidone iodine (PVPI) for ocular surface asepsis is widespread, both for surgical procedures and intravitreal injections [1–5]. Surgeries for cataracts, glaucoma, and intravitreal injections have become common and frequent procedures in ophthalmology, leading to the serial exposure of patients’ eyes to iodine solutions [5–10]. These changes are directly associated with dry eye syndrome [11, 12].

In particular, studies have demonstrated that intravitreal injections used to treat diabetic macular edema or age-related macular degeneration result in significant changes in the ocular surface, leading to dry eye syndrome and damage to homeostasis of the ocular surface [5, 11, 13, 14].

Dry eye syndrome is a multifactorial disease of the ocular surface characterized by the loss of tear film homeostasis, hyperosmolarity, inflammation, damage and neurosensory abnormalities [11, 15–18]. Its etiology is variable, ranging from nonspecific inflammation of the ocular surface to direct chemical or physical aggression, infections, and autoimmune diseases [11, 12, 15, 19].

In addition to the most common symptoms, burning sensation, itching, speck, eye redness, excess tearing reflex, brightness sensitivity, and quality of vision loss are also frequent findings that affect efficiency at work and the quality of life of patients [11, 12, 15, 19, 20].

This study aimed to observe changes in the ocular surface and tear film due the serial use of 2% PVPI, the gold standard drug for asepsis of the ocular surface. As it is well known that pre-injection antisepsis of the ocular surface with PVPI has a toxic effect on the corneal epithelium, the aim is to identify changes in the tear film and ocular surface and avoid serious problem like dry eye syndrome [11, 12, 21, 22].

## Methods

A case-control study was conducted at the Ophthalmology and Psicology Clinic (APMTO MD) in Londrina, Paraná. The patients were recruited from the Retina and Vitreous Institute (AMBC MD) in Londrina, Paraná. The study included 34 individuals (68 eyes). 14 males, 20 females, aged 48 to 94 years. All participants signed the informed consent form, which allowed their participation in the study. Inclusion criteria were individuals who received application of 2% PVPI eyedrops for anti-VEGF IVIs treatment with the contralateral eye used as control, that had not been treated with any topical medication during the same period of applications and good comprehension of the Ocular Questionnaire Surface Disease Index (OSDI). Exclusion criteria were patients who could not understand the OSDI questionnaire; patients using antidepressant medicine, diuretics, sympathomimetics, eye drops for glaucoma, or eye lubricants; people with allergies to iodine; unfavorable clinical conditions to undergo the examination procedures for the study; inappropriate test quantity and quality; unsatisfactory images or unsatisfactory and inadequate data.

The study was approved by the Ethics and Research Committee Involving Human Beings of the State University of Londrina by N. 5.300.176.

The individuals underwent directed clinical and ophthalmological analysis, received explanations about the study, used their data, and signed consent forms. All clinical measures were performed using the IDRA equipment (SBSSISTEMI, Orbasano, Torino, Italy), at which time the OSDI questionnaire was also applied and tear osmolarity was collected using the I-PEN^®^ (I-MED PHARMA INC. Dollard-des-Ormeaux, QC, Canada). All examinations and administration of the questionnaire were performed by the same professional. No drops or medications that could cause changes in any subsequent measurements were used.

The variables analyzed were age, sex, date of the last PVPI application, number of PVPI applications, OSDI questionnaire, tear osmolarity, NBUT, tear film interferometry, tear meniscus height, percentage of meibomian gland loss, and blink quality. The sequence of procedures obeyed the following order: Explanation to the subject regarding the exams and questionnaire to which he would be submitted, guidance to the patient not to identify in any way the eye being treated and the eye not treated during data collection, nor during the questionnaire OSDI; patient positioning in the IDRA® equipment; capture of blinking quality video images; capture of tear film interferometry; capture of images to measure the height of the tear meniscus and immediate measurement; capture of tear film (NBUT); image capture for the percentage of meibomian gland loss by everting the lower eyelid with a cotton swab; positioning the patient outside the IDRA equipment; application of the I-PEN® electrode to capture tear osmolarity in the lower conjunctiva, first in the right eye, and subsequently in the left eye; application of the OSDI questionnaire.

All statistical analyses were performed using STATA® 18.0 Software and p-values ≤ 0.05 indicated statistical significance.

The Shapiro-Wilk test was used to verify data normality. Data that did not follow a normal distribution were analyzed using the Wilcoxon rank-sum test and were described as means and as medians and interquartile ranges. Data that showed normality were analyzed using the Student’s T test and presented as means and standard deviations. Descriptive, quantitative, and multivariate analyses compared treated (case) and untreated (control) eyes.

## Results

The average number of IVIs in treated eyes was 12 (range 6–20). The results in treated eyes compared with untreated eyes were respectively: median OSDI 16 (IQR 6–39) and 12.5 (IQR 8–39) (*p* = 0.380); mean NIBUT 10.30 (SD ± 2,62) and 10.78 (SD ± 2.92) ( s, *p* = 0.476); median blinking quality 100 (IQR 100) and 100 (IQR 100 ) (%, *p* = 0.188); median lipid layer 87 (IQR 77–90) and 86 (IQR 74–100) (nm, *p* = 0.451); median meniscus height 0,22 (IQR 0.19–0.31) and 0.24 (IQR 0.20–0.27) (mm, *p* = 0.862), median Meibomian gland athrophy 33 (IQR 24–45) and 31.5 (IQR 25–39) (%, *p* = 0.524); and mean osmolarity 306.6 (SD ± 21.13) and 313.8 (SD ± 29) (mOsm, *p* = 0.297).). The results revealed that the use of 2% PVPI did not affect the analyzed variables in a statistically significant way. All data is summarized on Table 1.

**Table 1:** Differences in ocular surface parameters between treated and fellow eyes

	Treated eyes	Fellow eyes	*n*	*p*-value
Days of last application^↑^	52 (28–184)	0	34	
Number of application^↑^	12 (6–20)	0	34	
OSDI^↑^	16 (6–39)	12.5 (8–39)	68	0.830
NBUT* (s)	10.30 (2.62)	10.78 (2.92)	68	0.476
Blink quality^↑^ (%)	100 (100)	100 (100)	68	0.188
Lipid layer^↑^ (nm)	87 (77–90)	86 (74–100)	68	0.451
*Meniscus* high^↑^ (mm)	0.22 (0.19–0.31)	0.24 (0.20–0.27)	68	0.862
Meibomian gland loss^↑^ (%)	33 (24–45)	31.5 (25–39)	68	0.524
Osmolarity* (mOsm)	305.6 (21.13)	313.8 (29)	40	0.296

*Mean values with standard deviations (SD) and ^↑^ median values with interquartile range (IQR). NTBUT = non invasive tear film break up time. N = number of eyes

These results are disposable on fig 1, 2, 3, 4, 5, 6, 7, 8 and 9 as annexed.

**Fig. 1:**
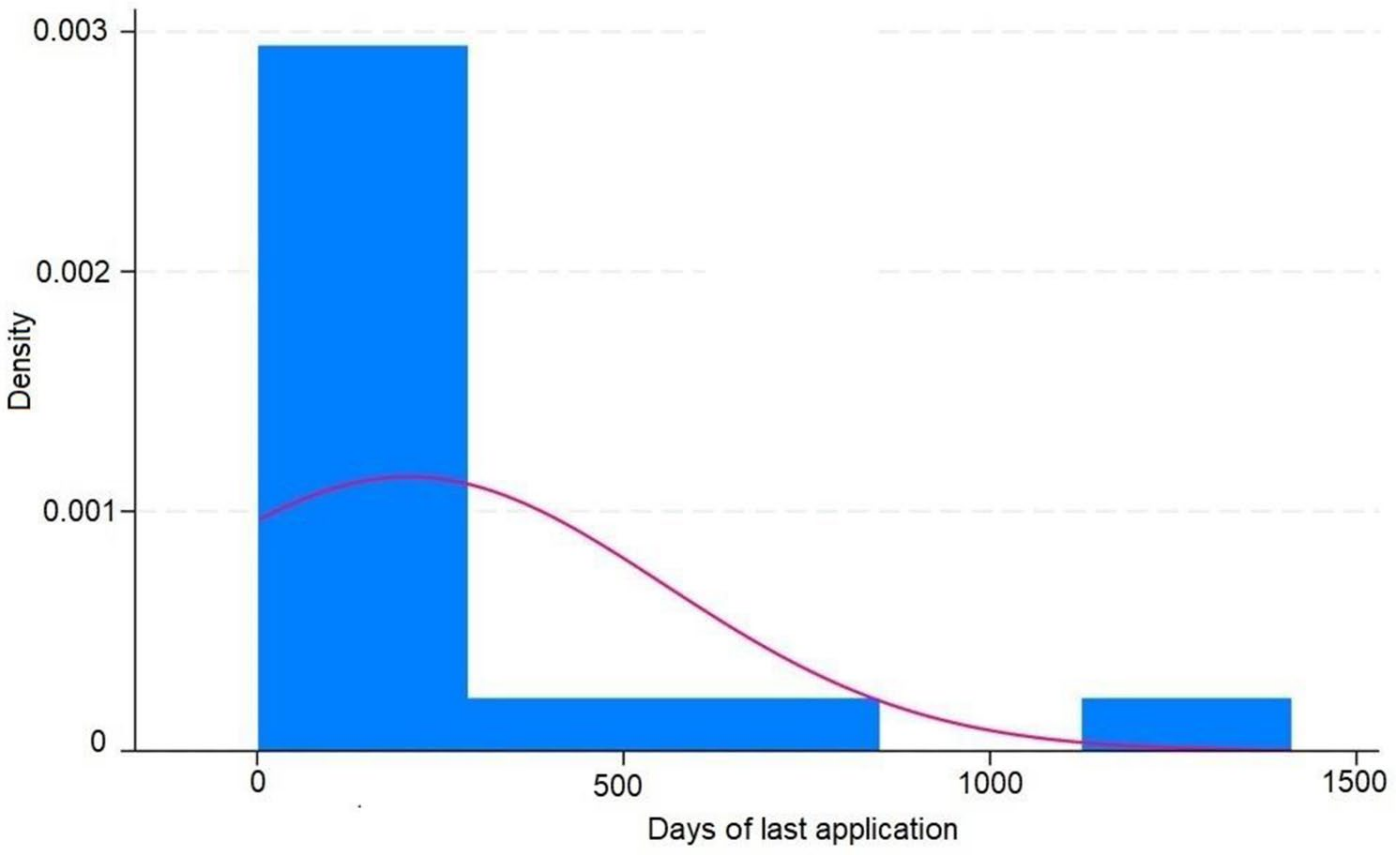
Histogram showing the days of last application of IVIS ( intravitreal injections ) in treated eyes and the density showing the proportion of eyes in each period of time

**Fig. 2:**
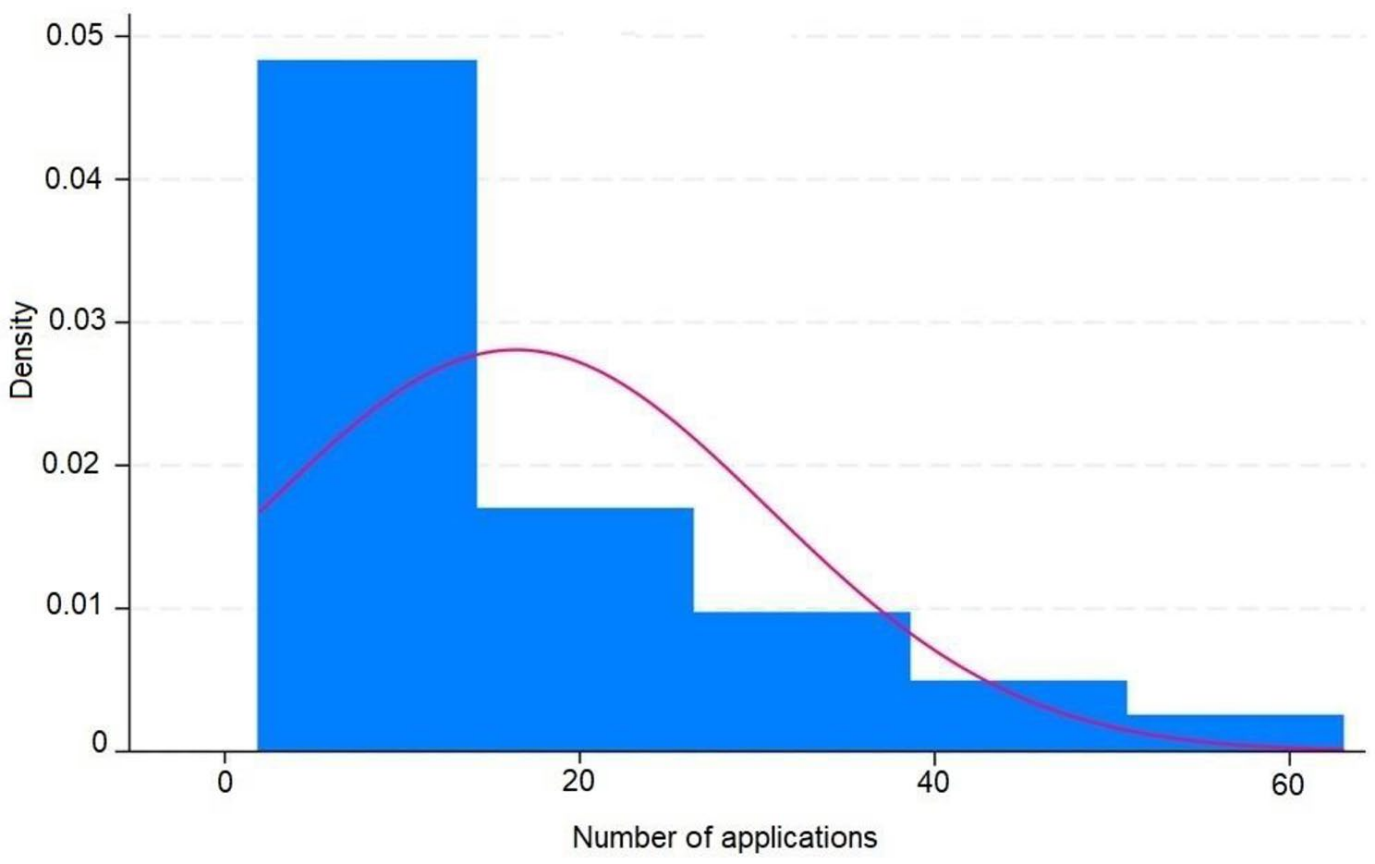
Histogram showing the number of application ov IVIS ( intravitreal injections ) in treated eyes and the density showing the proportion of eyes in each amount of number of applications

**Fig. 3:**
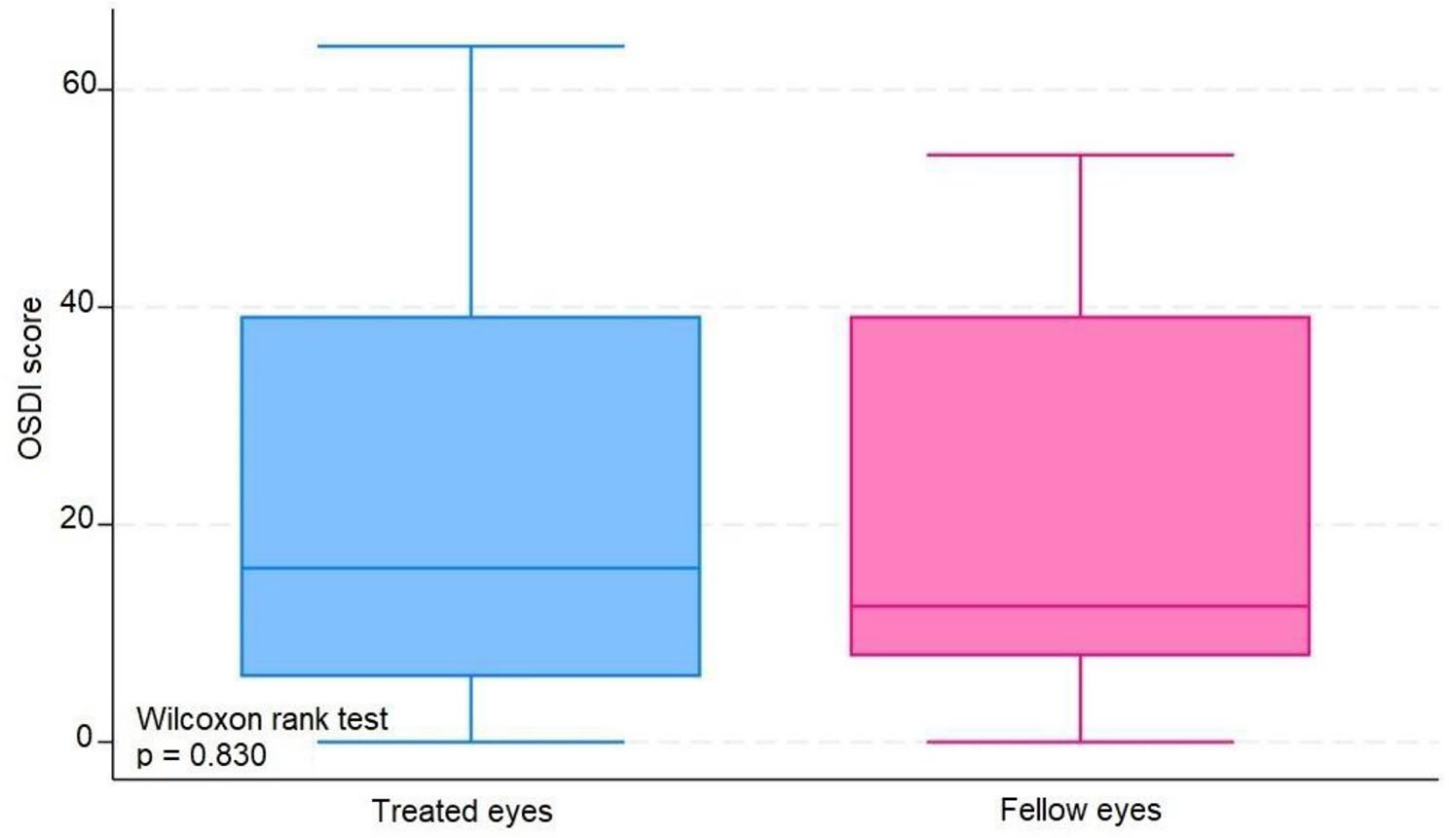
Blue box plot showing score OSDI ( Ocular Surface Disease Index ) in treated eyes comparing with pink box plot showing score OSDI in fellow eyes

**Fig. 4:**
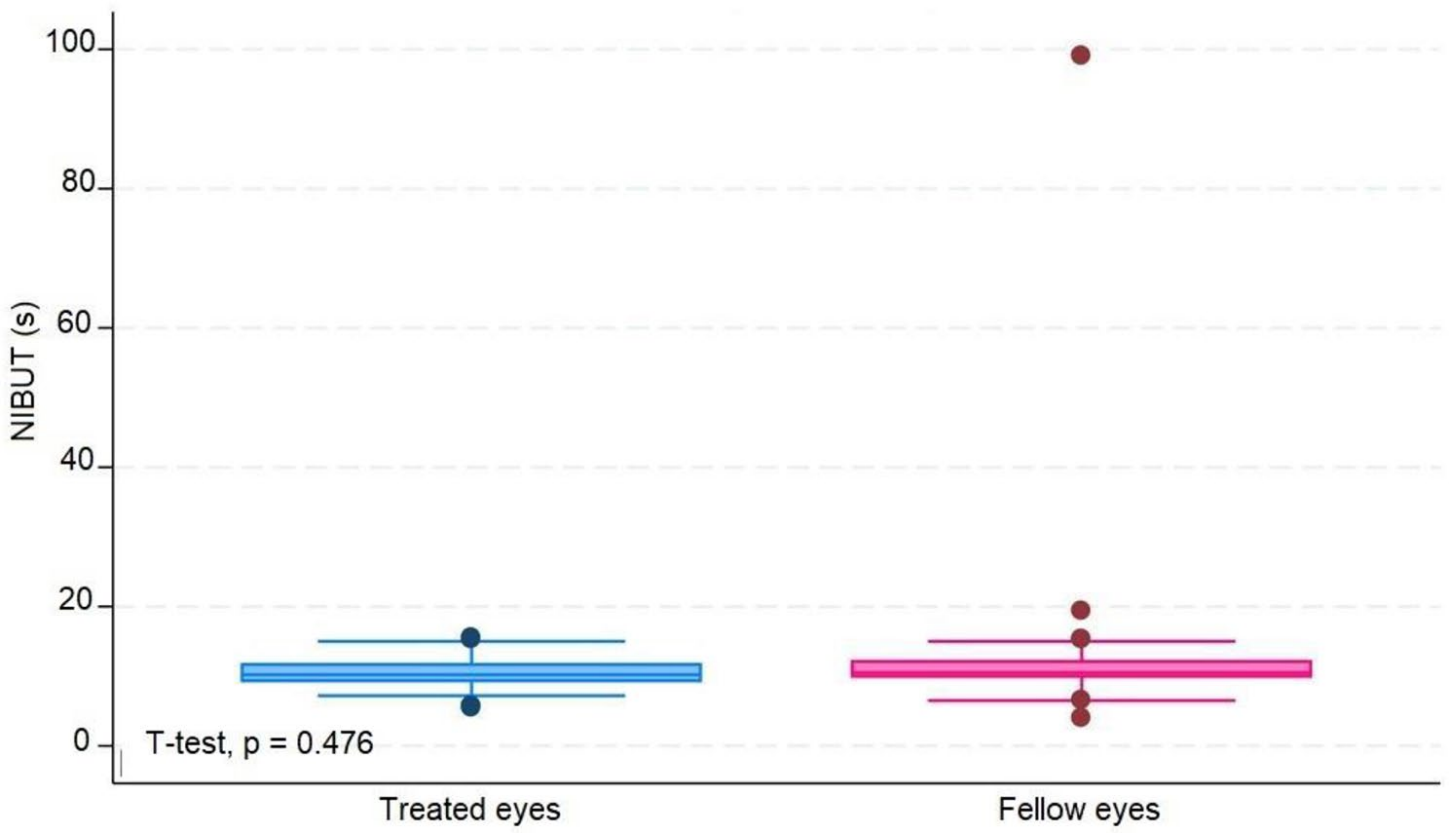
Blue box plot showing NIBUT ( non invasive break up time ) in seconds in treated eyes comparing with pink box plot showing NIBUT in seconds in fellow eyes

**Fig. 5:**
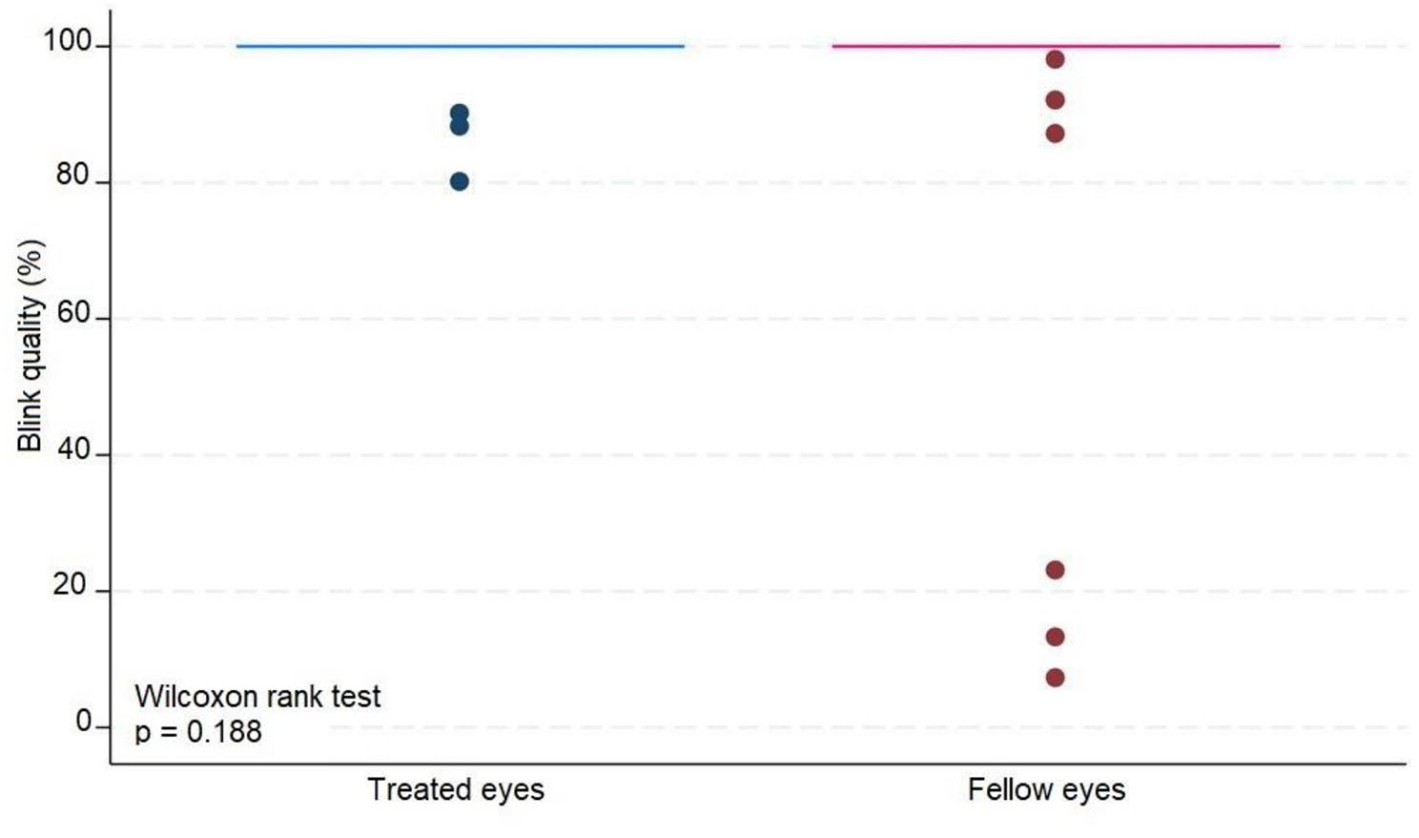
Blue box plot showing blink quality in treated eyes comparing with pink box plot showing blink quality in fellow eyes

**Fig. 6:**
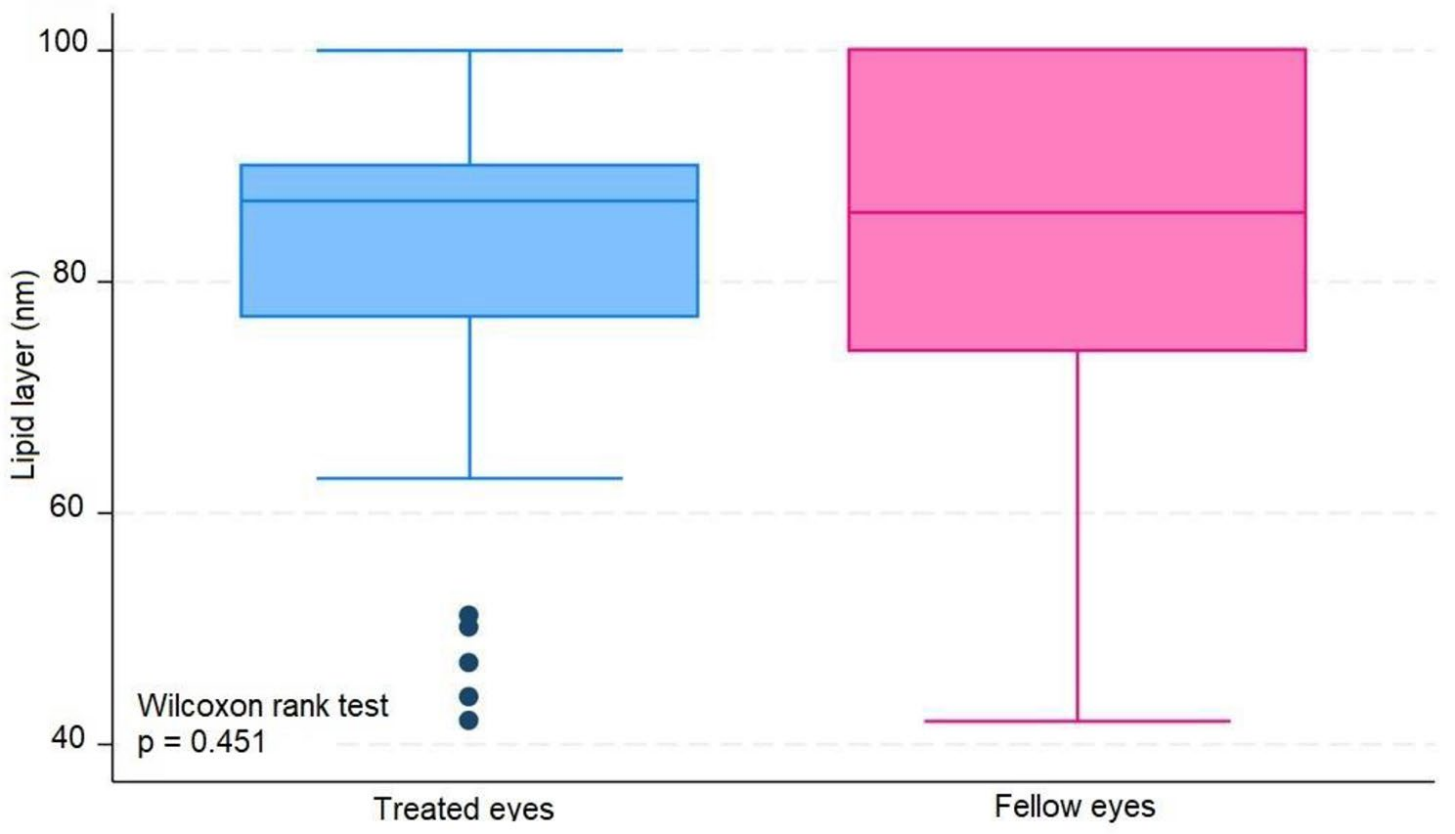
Blue box plot showing lipid layer in treated eyes comparing with pink box plot showing lipid layer in fellow eyes

**Fig. 7:**
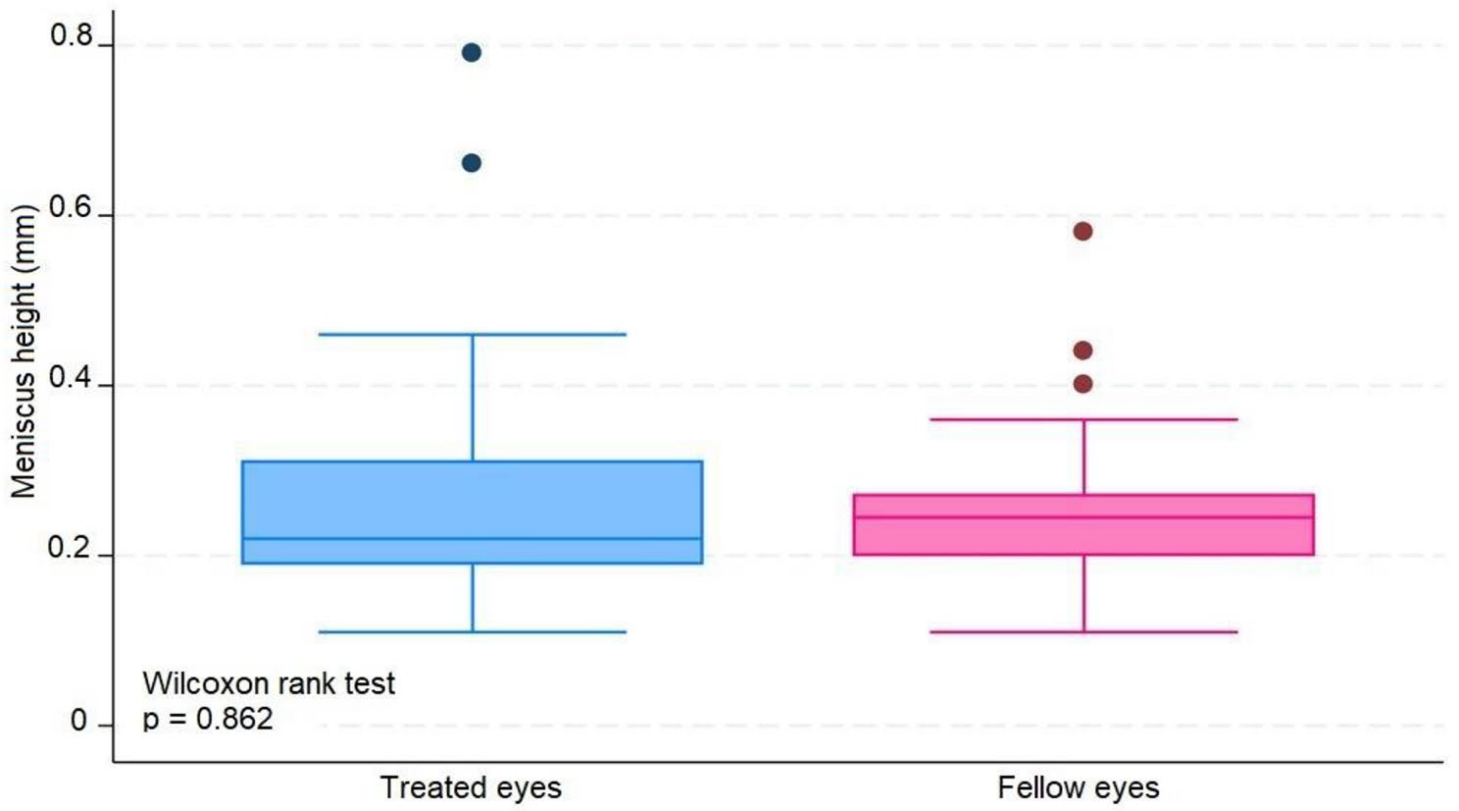
Blue box plot showing meniscus height in milimeters in treated eyes comparing with pink box plot showing meniscus height in milimeters in fellow eyes

**Fig. 8:**
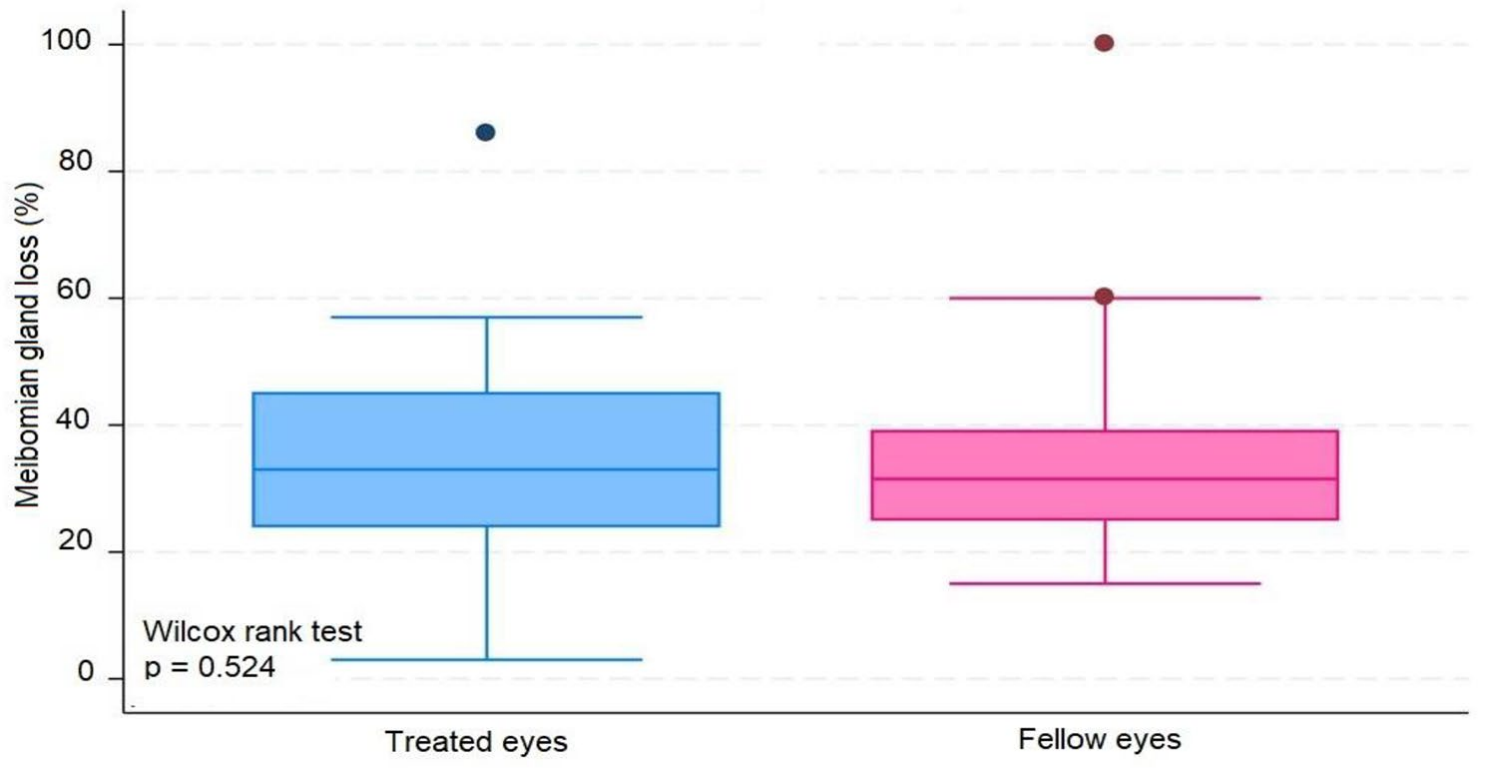
Blue box plot showing Meibomian gland loss in treated eyes comparing with pink box plot showing Meibomian gland loss in fellow eyes

**Fig. 9:**
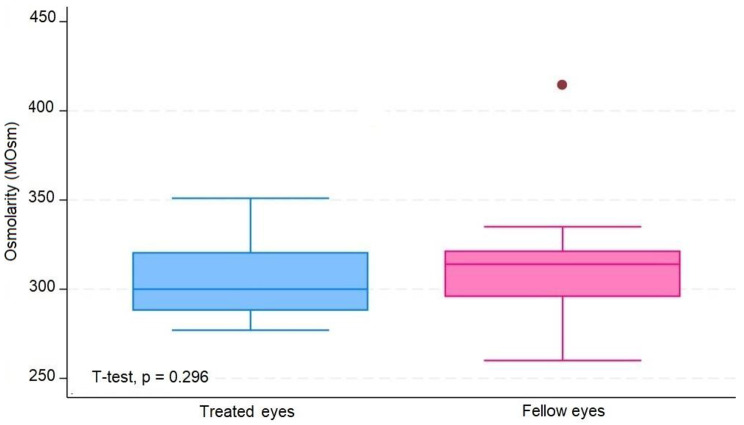
Blue box plot showing tear osmolarity in miliosmoles in treated eyes comparing with pink box plot showing osmolarity in miliosmoles in fellow eyes

Through multivariate analysis, we obtained some interesting outcomes as follows:

When controlling for NIBUT, meibomian gland atrophy, number of applications, and days of the last application according to treatment, sex was an important variable in explaining the variability in the OSDI score (coef = 15.63 | p-value = 0.003). On average, controlling for the abovementioned variables, being female contributed to an increase in the OSDI to 15.63 points.

After controlling for meniscus height and age according to treatment, tear osmolarity contributed significantly to variability in the lipid layer (coef = -0.266, *p* = 0.004). In this sense, the addition of one unit in tear osmolarity led to a -0.266 drop in the lipid layer.

After controlling for meniscus height, OSDI, days since the last application, age, and sex according to the treatment, these factors contributed significantly to the variability in the lipid layer [(coef = 0.562 | p-value = 0.004) (coef = − 5.622 | p-value = 0.048)]. In this sense, the addition of one year of age led to a decrease of -0.562 on average. For the same treatment group, female sex led to a decrease of -5,622.

Age, lipid layer, meniscus height, sex according to treatment, age according to treatment, and sex were important factors for explaining the variability in tear osmolarity.

We noticed that a greater age correlated with lower tear osmolarity. However, being in the treated group reduced the decrease in tear osmolarity with advancing age.

Being female implied higher tear osmolarity. However, the increase in tear osmolarity was smaller in the treated group.

A greater height of the lipid layer and meniscus correlated with lower tear osmolarity.

## Discussion

The present study showed that the use of topical PVPI at 2% did not cause significant damage to the ocular surface when the findings of the ocular surface and tear film analyses were used.

Our results contradict some existing data indicating the toxicity of long-term iodine use on the ocular surface; we found two statistically relevant results that the application of iodine may improve the stability of the tear film in the elderly and women, since the eyes in older individuals and female patients that received iodine showed a smaller increase in tear osmolarity [4, 14, 23, 24].

Moreover, the results of this study corroborated some hypotheses that the use of PVPI could be positive in some dry eye disease diagnostic features, such as the improvement of the tear film meniscus height and the decrease of the tear film osmolarity [25, 26].

A localized anti-inflammatory surface effect of the anti-VEGF agent used in intravitreous injections should be considered and assessed in further studies [22, 25].

The literature review also shows that there was an improvement in the tear function of some patients who used iodine in ocular asepsis [25–27], perhaps due to an antimicrobial action preventing the proliferation of bacterial flora that could produce harmful enzymes or cause meibomitis and blepharitis [25, 26, 28].

The cell regeneration mechanism might have satisfactorily recomposed the ocular surface or the tear homeostasis might have compensates for the damage caused by iodine in the cells in question; furthermore, these are just hypotheses.

We also determined that the risk factors for dry eye disease, age and female sex [10, 16, 29, 30], were associated with the observed clinical data: greater ages lower the height of the tear meniscus, the greater the tear osmolarity, and the smaller the lipid layer of the tear film. The female sex was also associated with higher OSDI scores and fewer tear film lipid layers.

Regardless of the cause or consequence, the osmolarity and lipid layer of the tear film were inversely proportional.

Through multivariate analysis, we determined that the risk factors for dry eye syndrome, age, and female sex correlated with worse results in the tear meniscus measurement tests, OSDI questionnaire, and tear film interferometry, corroborating the literature implicating them as risk factors for dry eye disease [20, 30, 31].

Moreover, due to the sample size, false negatives, or simply because in practice, iodine in the amount and frequency used does not lead to histological damage that may reflect functional changes. The results did not discourage the use of iodine for ocular asepsis but also did not indicate its use for protocols with higher concentrations or more applications than those used in current protocols.

The strengths of the study are as follows: the same patient was the control and treated group, avoiding any environmental or medical bias. The number of injections administered was higher than that reported in other studies. No drops were used during the examination to avoid artificial changes to the tear film.

The limitations of this study were as follows: the study had a small sample size of 34 patients, resulting in 68 eyes being analyzed, which may have caused an analysis bias when using these data in the general population. We must remember that the analyzed population was from southern Brazil and had mostly descended from Italian, German, Spanish, and Portuguese immigrants; therefore, these data may only reflect the specific epidemiology of this population. The meibomian glands analyzed were located in the inferior tarsus.

## Conclusions

The use of iodine on the ocular surface was not significantly associated with any of the evaluated parameters. There were no statistically significant correlations between the tests applied to the case eyes. The current study indicates that the application of 2% topical povidone-iodine (PVPI) does not inflict significant damage to the ocular surface, as evidenced by the analyses of the ocular surface and tear film. Notable strengths of this study include the use of the same patient as both the control and the treated subject, which minimizes potential biases from environmental or medical factors. Additionally, the absence of any artificial agents during the examination ensures that the tear film remains unaltered.

Contrary to previous concerns regarding the long-term toxicity of iodine on the ocular surface, our findings suggest potential benefits of iodine application in stabilizing the tear film, particularly in older individuals and female patients. This is supported by a smaller increase in tear osmolarity in these groups following iodine application. Furthermore, the study corroborates hypotheses that PVPI may positively affect certain Dry Eye Disease diagnostic features, such as improved tear film meniscus height and reduced tear film osmolarity.

## Data Availability

No datasets were generated or analysed during the current study.

## Abbreviations

PVPI: Povidine or polyvinylpyrrolidone-iodine
IVIS: Intravitreal injections
VEGF: Vascular endothelial growth factor
OSDI: Ocular Surface Disease Index
NIBUT: Non invasive break up time
BQ: Blink quality
LL: Lipid layer
SD: Standard deviation
IQR: Interquartile range
NM: Nanometer
MM: Miliimeter
mOsm: Miliosmol
